# Association Between Magnesium Levels and Periodontal Disease: A Systematic Review

**DOI:** 10.7759/cureus.88744

**Published:** 2025-07-25

**Authors:** Tomas Cruz-Velasquez, Alain Raimundo Rodríguez-Orozco

**Affiliations:** 1 Institute of Chemical Biological Research, Michoacan University of San Nicolas de Hidalgo, Morelia, MEX; 2 Faculty of Medical and Biological Sciences, Michoacan University of San Nicolas de Hidalgo, Morelia, MEX

**Keywords:** inflammation, magnesium, mg/ca ratio, micronutrients, periodontal disease, periodontal health status, periodontitis, systematic review

## Abstract

Periodontal disease, a persistent inflammatory disorder that damages tissues supporting the teeth, is mainly triggered by an imbalanced microbial biofilm and an overactive immune-inflammatory reaction. Recent studies underscore the possible contribution of micronutrient shortages, especially in magnesium, in the progression of periodontitis. This systematic review synthesizes evidence on the association between magnesium levels, the magnesium/calcium (Mg/Ca) ratio, and periodontal disease, focusing on blood-based measures and dietary intake. Following the Preferred Reporting Items for Systematic Reviews and Meta-Analyses guidelines, we searched PubMed, Cochrane Library, and Google Scholar from inception to May 2024, including 10 observational and experimental studies in adults. Narrative synthesis revealed consistent inverse associations: higher magnesium levels correlated with reduced probing pocket depth, clinical attachment loss, and periodontitis prevalence, while favorable Mg/Ca ratios protected against progression (e.g., odds ratio of 6.28 for low ratios). Systemic associations, particularly with diabetes, showed lower magnesium in comorbid cases, with post-treatment improvements suggesting anti-inflammatory benefits. Heterogeneity precluded meta-analysis. These findings highlight the protective role of magnesium, warranting large-scale trials to guide supplementation and dietary strategies for periodontal management.

## Introduction and background

Periodontitis is a chronic inflammatory disease causing destruction of tooth-supporting structures [1]. Key features include periodontal pockets, clinical attachment loss (CAL), gingival bleeding assessed by probing pocket depth (PPD), and alveolar bone loss visible on radiographs [2]. If left untreated, periodontitis can result in tooth loss [3]; however, it is largely preventable and treatable [4]. Periodontitis is a widespread and multifaceted condition, triggered by a dysbiotic plaque biofilm and primarily advanced through an exaggerated host immune-inflammatory response. This progression is influenced by genetic factors, lifestyle choices, environmental conditions, and medication effects, all of which can act as contributing factors in the onset and advancement of periodontitis in susceptible individuals [5,6]. Additionally, micronutrient deficiencies may also contribute to this disease pathway, with their effects being modulated by genetic polymorphisms related to nutrient metabolism, as well as individual behaviors and lifestyle factors [7].

Thus, the standard periodontal therapy employed is scaling and root planing (SRP), a non-surgical treatment designed to meticulously clean the teeth and root surfaces, removing plaque, calculus, and bacterial endotoxins. Its primary objectives are to reduce periodontal pocket depths, promote reattachment of gingival tissue to the tooth surfaces, and halt the progression of periodontal disease [4,8]. However, recent research has highlighted the importance of systemic factors, including nutrition, in periodontal disease [9]. The role of nutrients, especially micronutrients such as magnesium, has gained attention due to their impact on the immune system and inflammation [7].

Magnesium, among other nutrients, is essential for various bodily functions, including enzyme activity and immune response [10-12]. Nutrient deficiencies, including magnesium depletion, can exacerbate periodontal disease and affect overall health. Understanding how different nutrients influence periodontal inflammation can help identify nutritional risk factors and adapt prevention and treatment strategies for individuals with specific deficiencies [7,9]. Magnesium is a crucial element in the body, serving as a cofactor for over 600 enzymes involved in energy metabolism, protein and nucleic acid synthesis, and potassium and calcium transport [13-15]. Approximately 60-65% of the body’s magnesium resides in bones, with 27% in muscles, and about one-third of bone magnesium is exchangeable to maintain normal extracellular concentrations (0.7-1.0 mmol/L) [7,16-18]. In serum, 32% is bound to albumin, and 55% exists as free ionized magnesium, the bioactive form. Although clinical deficiency is rare due to renal regulation, subclinical deficiency is common and often unnoticed, potentially impacting periodontal disease by affecting inflammation and immune response [10,15,16]. Serum magnesium homeostasis is primarily maintained through kidney function, ensuring stable levels even with low dietary intake by reducing renal excretion and releasing magnesium from bone reserves [7,13,17].

Inadequate magnesium intake, even at subclinical levels, may contribute to persistent low-grade inflammatory stress, potentially increasing the risk of chronic diseases [19]. Research has demonstrated strong correlations between insufficient magnesium levels and conditions such as diabetes mellitus, metabolic syndrome, cardiovascular diseases, hypertension, and pregnancy-related complications [20-22]. Studies exploring magnesium deficiency have revealed intricate proinflammatory mechanisms involving calcium and N-methyl-D-aspartate pathways, which elevate proinflammatory cytokines, stimulate reactive oxygen species in endothelial cells and leukocytes, and amplify the release of inflammatory cytokines and acute-phase proteins such as C-reactive protein (CRP) [7,23,24]. This inflammatory environment can worsen periodontal disease by promoting the breakdown of gum tissues and bone, suggesting that the role of magnesium in inflammation and systemic health could directly impact periodontal health. Magnesium deficiency decreases osteoblast numbers and osteoclast activity, causing aplastic bone disease and low bone mass [11,16,17]. In vitro, magnesium deficiency reduces osteoblast proliferation, leading to inadequate bone formation and poor resorption regulation, resulting in bone loss [25]. This suggests that magnesium’s role in systemic health impacts periodontal health.

With this background, this systematic review focuses on the association between magnesium levels, the magnesium/calcium (Mg/Ca) serum ratio, and periodontal disease, being the first of its kind to our knowledge. Although the effects of various macro and trace elements in saliva and gingival crevicular fluid (GCF) related to periodontal disease have been studied, there are no related articles comprehensively summarizing the role of serum/plasma magnesium levels and dietary magnesium intake in maintaining periodontal health. Therefore, this review addresses a critical gap in the literature by synthesizing available evidence, highlighting magnesium’s potential as a modifiable risk factor that influences inflammation, bone homeostasis, and immune responses, which could inform nutritional interventions, enhance preventive strategies, and improve clinical outcomes for periodontal disease management.

## Review

Methodology

**Figure 1:**
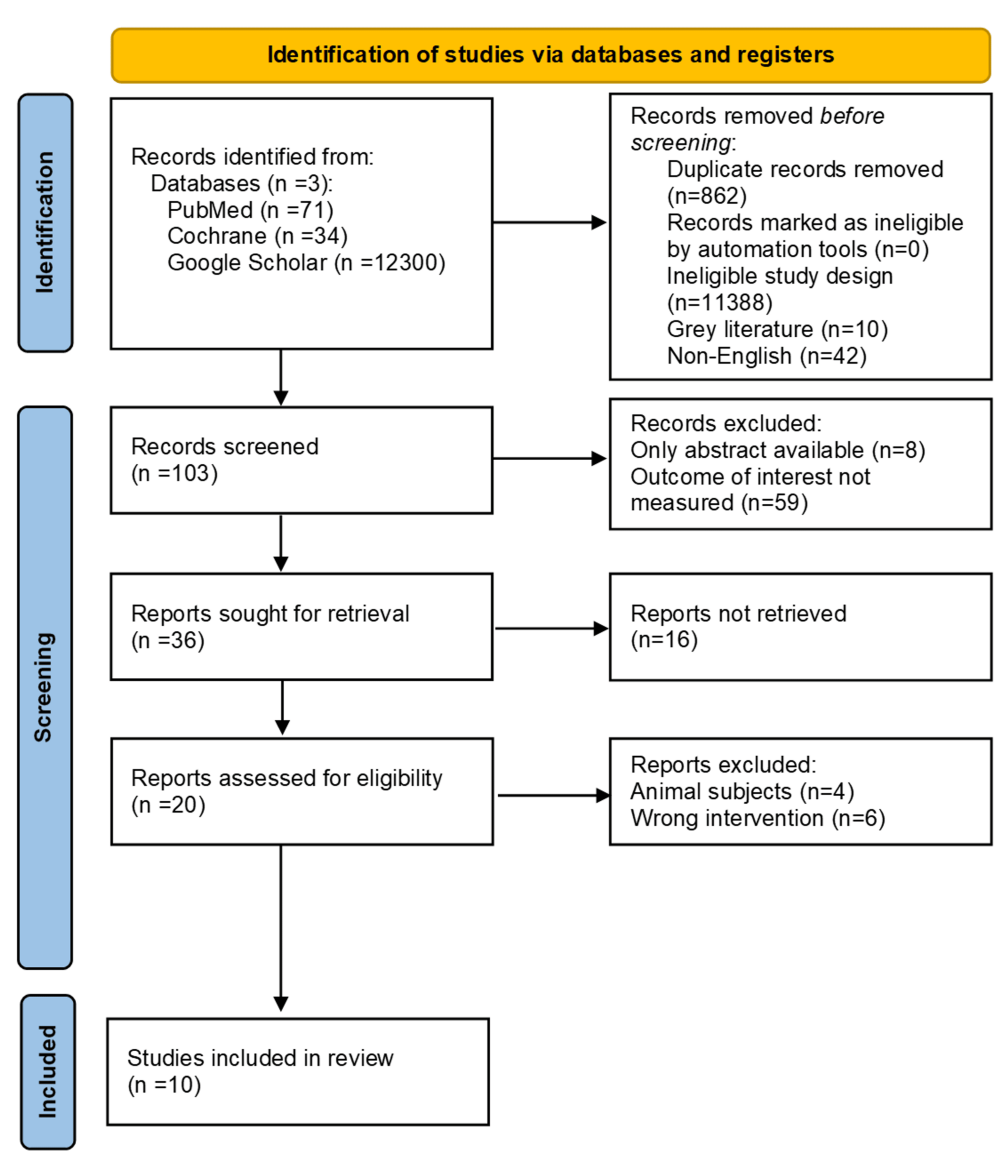
Preferred Reporting Items for Systematic Reviews and Meta-Analyses (PRISMA) flow diagram illustrating the results of literature search and study selection.

This systematic review adhered to the Preferred Reporting Items for Systematic Reviews and Meta-Analyses (PRISMA) 2020 guidelines [26] to ensure transparency and reproducibility. The PRISMA flow diagram is presented in Figure 1.

Research Question

This systematic review was designed to address the following PECO (Population, Exposure, Comparator, Outcome) question: “In adults, do altered magnesium levels, an imbalanced Mg/Ca serum ratio, or inadequate magnesium dietary intake (compared to normal magnesium levels, a balanced Mg/Ca serum ratio, and adequate magnesium dietary intake) affect the prevalence, incidence, or severity of periodontal disease?”

Population (P): Adults (in general population or clinical settings), including those with and without periodontal disease, to enable comparative analyses.

Exposure (E): Altered magnesium levels, imbalanced Mg/Ca serum ratio, or inadequate magnesium dietary intake.

Comparator (C): Normal magnesium levels, balanced Mg/Ca serum ratio, and adequate magnesium dietary intake.

Outcome (O): Prevalence, incidence, or severity of periodontal disease (e.g., periodontal pocket depth, CAL, and inflammatory markers).

Eligibility Criteria

Studies were included if they were peer-reviewed, English-language articles examining the association between magnesium levels (serum/plasma or dietary intake) and periodontal disease in adults. Exclusion criteria encompassed in vitro studies, animal studies, editorials, clinical case reports, literature reviews, and non-English articles. Gray literature (e.g., reports, theses, and conference papers) was excluded to prioritize high-quality, peer-reviewed evidence. Additionally, studies analyzing magnesium levels in saliva or GCF were excluded to focus solely on blood-based measures (serum and/or plasma), as these aligned with our PECO framework.

Information Sources

A comprehensive literature search was conducted across electronic databases, including PubMed, Cochrane Library, and Google Scholar, from inception to May 2024.

Search Strategy

The search strategy employed keywords and Medical Subject Headings (MeSH) terms such as “magnesium,” “periodontitis,” “periodontal disease,” and “inflammation,” combined with Boolean operators (AND/OR) to enhance relevance and sensitivity. Specific search strings included: “magnesium” AND “periodontitis”; “magnesium levels” AND “periodontitis”; “magnesium” AND “periodontal disease”; and “magnesium” AND “inflammation” AND “oral health.” Adjustments were made as needed to refine results. No language filters were applied initially, but only English-language articles were retained during screening.

Study Selection

Two independent reviewers screened titles and abstracts for initial eligibility, followed by full-text review of potentially relevant articles. Titles without abstracts but appearing relevant were retrieved for full-text assessment. Disagreements on inclusion were resolved through discussion and consensus.

Data Collection Process

Data were independently extracted by two reviewers from eligible studies, focusing on study characteristics (e.g., design, population, setting), exposure details (e.g., magnesium measurement methods), comparators, and outcomes. Quantitative data included means, standard deviations, group differences, correlation/regression coefficients, incidence rate ratios (IRRs), odds ratios (ORs), and 95% confidence intervals (CIs), along with p-values. Discrepancies in data extraction were resolved by consensus.

Risk of Bias in Individual Studies

Methodological quality was assessed independently by two reviewers using design-specific tools. Observational studies were evaluated for selection bias, information bias, confounding, and follow-up adequacy (e.g., using the Newcastle-Ottawa Scale). Randomized controlled trials were assessed for randomization, blinding, and allocation concealment (e.g., using the Cochrane Risk of Bias Tool). Risk was categorized as low, moderate, or high, with disagreements resolved through discussion.

Synthesis Methods

Due to heterogeneity in study designs, interventions, settings, and outcome measures, a meta-analysis was not feasible, and a narrative synthesis was conducted instead. Results were qualitatively summarized by grouping findings according to exposure type (e.g., serum magnesium levels vs. dietary intake) and outcome metrics (e.g., periodontal pocket depth, CAL), with an emphasis on emerging patterns in associations and their clinical implications.

Results

Study Characteristics

In total, 10 studies were identified as relevant for investigating the role of magnesium in periodontal health. These included five cross-sectional studies [27-31], one cohort study [32], two case-control studies [33,34], and two experimental studies [35,36] (Table 1). Most studies assessed magnesium through blood measurements, while two also evaluated dietary intake [27,31]. Additionally, two studies examined the Mg/Ca serum ratio [27,28], and one focused on the Ca/Mg ratio [32], highlighting potential interactions between these minerals.

**Table 1:** Characteristics of the included studies. SHIP: Study of Health in Pomerania; Mg: magnesium; Ca: calcium; Zn: zinc; Cu: copper; Fe: iron; DM2: type 2 diabetes mellitus; SRP: scaling and root planing; BoP: bleeding on probing; GI: gingival index; AAP 1999: American Academy of Periodontology, 1999 classification of periodontal diseases; NHANES: National Health and Nutrition Examination Survey

Author	Year	Country	Study type, sample	N	Age (y)	Dietary intake assessment	Nutritional status assessment	Periodontal status	Association assessment	Main results/Conclusions
Meisel et al. [27]	2005	Germany	Cross-sectional, SHIP participants	4,290	20–80 years	Intake of Mg-containing drugs	Serum Mg/Ca (quartiles)	%PPD ≥ 4 mm, %CAL > 4 mm, number of teeth	Adjusted OR (univariate and multivariate logistic regression)	Inverse relationship between serum Mg/Ca ratio with %PPD ≥ 4 mm and %CAL > 4 mm in individuals aged ≥40 years (lowest quartile vs. highest quartile). Individuals using Mg-containing drugs presented reduced CAL
Yoshihara et al. [32]	2011	Japan	Cohort (6 years), Nigata city inhabitants	309	73 years	__	Serum Ca/Mg (quartiles)	Periodontal disease events (CAL ≥ 3 mm per year at any tooth site)	Adjusted OR (multiple logistic regression)	The Ca/Mg ratio negatively correlated with periodontal disease events in smokers
Pushparani et al. [33]	2014	India	Case-control, specialty hospital and dental college patients	600	25–55 years	__	Serum levels of Mg, Zn	DM2 with periodontitis, DM2 without periodontitis, only periodontitis (mean PPD ≥ 5 mm, mean CAL ≥ 3 mm in at least 40% of teeth) vs. healthy	Differences among groups	Negative association of serum Mg with CAL, only in DM2 patients with periodontitis
Meisel et al. [28]	2016	Germany	Cross-sectional SHIP-1 participants	3300	20–80 years	__	Serum Mg/Ca (quartiles)	%PPD ≥ 4 mm, %CAL > 4 mm, number of teeth	Multivariate regression (generalized linear models)	Higher ratios of Mg/Ca were associated with improved CAL. Attachment level gain increased with high Mg (positive β coefficients)
Shetty et al. [35]	2016	India	Experimental, Institute of Dental Science subjects	120	25–60 years	__	Serum levels of Mg	DM2 with periodontitis, DM2 without periodontitis, only periodontitis (mean PPD ≥ 5 mm, mean CAL ≥ 3 mm in at least 40% of teeth), vs. healthy	Differences among groups	Baseline serum Mg levels were significantly lower in patients with periodontitis and in DM2 patients, both with and without periodontitis, compared to controls. Following treatment (21 days after SRP), a significant increase in mean serum Mg levels was observed across all three test groups in comparison to healthy controls
Sundaram et al. [36]	2017	India	Experimental, outpatients	120	__	__	Serum levels of Mg, Zn, Cu	Chronic periodontitis (PPD ≥ 5 mm, CAL ≥ 4 mm, BoP, number of teeth), chronic periodontitis with controlled DM2, and chronic periodontitis with uncontrolled DM2	Differences among groups	There was no significance of Mg at baseline and after treatment
Taru et al. [29]	2017	India	Cross-sectional, outpatients from a dental college and hospital	60	30–50 years	__	Serum Mg and Zn levels	Chronic periodontitis patient (mean PPD ≥ 5 mm, CAL ≥ 3 mm in at least 40% of teeth), vs. healthy	Differences between groups	Serum Mg levels were significantly lower in subjects with chronic periodontitis compared to healthy controls
Dannan and Hanno [30]	2018	Syria	Cross-sectional, dental clinic patients	29	__	__	Plasma levels of Mg and Cu	Periodontal disease (AAP 1999): Acute gingivitis, chronic gingivitis, chronic periodontitis, aggressive periodontitis	Differences between groups	Plasma Mg levels were significantly higher in patients with chronic and aggressive periodontitis compared to those with acute and chronic gingivitis
Li et al. [31]	2022	USA	Cross-sectional, 2013–2014 NHANES participants	3028	≥30 years	Dietary intake of Mg by 24-hour recall	Mg daily intake (quintiles)	Periodontitis: PPD ≥ 4 mm in ≥ 2 interproximal sites or one site with PPD ≥ 5 mm, CAL ≥ 3 mm in ≥ 2 interproximal sites (not on the same tooth)	Adjusted OR (multiple logistic regression)	Negative association between dietary Mg intake and prevalence of periodontitis
Raju et al. [34]	2023	India	Case-control, Dental College patients	110	35–65 years	__	Serum levels of Mg, Zn, Cu, Fe	Chronic periodontitis (PPD ≤ 5 mm, CAL 3-4 mm, GI > 2, number of teeth) vs. healthy (PPD ≤ 3mm, CAL 0mm, GI < 2)	Differences among groups	Serum Mg levels were significantly lower in subjects with chronic periodontitis compared to healthy controls

Main Findings

One notable cross-sectional study by Meisel et al. [27] examined individuals aged 20 to 80 years and revealed that a higher serum Mg/Ca ratio correlated with better periodontal outcomes, including reduced PPD (r = -36.1, p < 0.001), less CAL (r = -42.8, p = 0.001), and a greater number of remaining teeth (r = 7.5, p = 0.019). Extending this inquiry, the study also assessed magnesium-containing drug use among 180 participants aged 40 years and older, finding that those consuming such drugs exhibited lower CAL (2.7 ± 1.6 vs. 3.5 ± 1.6, p < 0.01) and PPD (2.4 ± 0.6 vs. 2.8 ± 0.9, p < 0.01) compared to matched controls (adjusted for age, sex, smoking, and education). These findings suggest that both endogenous magnesium ratios and supplemental intake may mitigate periodontal damage.

Building on their prior work, Meisel et al. [28] conducted a follow-up cross-sectional analysis within the Study of Health in Pomerania (SHIP-1), tracking 2,432 participants aged 20 to 80 years over five years to evaluate the long-term impact of baseline magnesium levels on attachment loss and tooth loss. After excluding edentulous individuals, those on diuretics, or with incomplete data from an initial 3,300 follow-up participants, the results showed that higher Mg/Ca ratios were associated with preserved attachment levels in a dose-dependent manner. Compared to the lowest quartile, the second and third quartiles demonstrated reduced attachment loss (ß = 0.12; 95% CI = 0.02-0.22, p = 0.021), with the strongest effect in the fourth quartile (ß = 0.24; 95% CI = 0.12-0.36, p < 0.001). Over the five-year period, attachment loss progressively decreased with rising Mg/Ca ratios, stabilizing to no further loss in the highest quartile. Furthermore, high Mg/Ca ratios offered protection against tooth loss, especially in those with systemic inflammation (CRP >3 mg/L), where elevated CRP increased tooth loss risk but was counteracted by higher Mg/Ca ratios. The study also found fewer individuals with elevated CRP in the highest Mg/Ca quartile, with a significant reduction in CRP risk for those with high levels at both baseline and follow-up (OR = 0.57, p = 0.003). This underscores magnesium’s anti-inflammatory potential in modulating periodontal progression.

Shifting to comparisons between diseased and healthy groups, Taru et al. [29] conducted a cross-sectional study of 60 participants aged 30 to 50 years (30 with chronic periodontitis and 30 healthy controls) to compare serum magnesium and zinc levels. They reported significantly lower mean serum magnesium in the periodontitis group (1.30 ± 0.78 mEq/L) versus controls (1.89 ± 0.48 mEq/L), with an unpaired Student’s t-test confirming statistical significance (p < 0.05). This alteration in magnesium levels among periodontitis patients points to its possible involvement in disease pathology.

In a related exploration of plasma levels, Dannan and Hanno [30] compared copper and magnesium in 29 patients with various periodontal conditions: eight with acute gingivitis, nine with chronic gingivitis, six with chronic periodontitis, and six with aggressive periodontitis. Magnesium levels differed significantly between acute (1.85 mg/dL) and chronic gingivitis (2.03 mg/dL; p < 0.05), as well as between chronic (2.25 mg/dL) and aggressive periodontitis (2.48 mg/dL; p < 0.05), indicating higher magnesium in more severe or aggressive forms. These variations suggest magnesium’s dynamic role across different stages of periodontal disease.

Expanding to dietary perspectives, Li et al. [31] analyzed retrospective data from the US National Health and Nutrition Examination Survey (NHANES) 2013-2014 to determine the relationship between dietary magnesium and periodontitis. Participants were divided into five groups (quintiles) based on their daily magnesium intake, with the prevalence of periodontitis being 49.5% in the lowest quintile (Q1), 41.2% in Q2, 39.3% in Q3, 42.2% in Q4, and 39.3% in the highest quintile (Q5). Across all models developed, there was a negative relationship between dietary magnesium intake and periodontitis prevalence. Logistic regression analysis showed that participants in the highest quintile of magnesium intake had a lower prevalence of periodontitis compared to those in the lowest quintile. In model 1, the OR was 0.65 (95% CI = 0.5-0.86), while in models 2 and 3, the OR was 0.69 (95% CI = 0.52-0.92). The trend was statistically significant in all models (p < 0.05). They concluded that dietary magnesium intake was negatively associated with the incidence of periodontitis. Given widespread magnesium deficiency in the United States, the authors proposed dietary increases as a strategy to reduce periodontitis incidence.

Transitioning to longitudinal evidence, Yoshihara et al. [32] followed 309 elderly smokers in Japan over six years in a cohort study, examining serum calcium, magnesium, and Ca/Mg ratios in relation to periodontal disease progression. An inverse dose-response emerged, with lower Ca/Mg ratios linked to higher odds of disease events: first quartile (OR = 6.28, 95% CI = 1.45-27.28, p = 0.014) and second quartile (OR = 5.96, 95% CI = 1.30-27.34, p = 0.022) versus the fourth. This highlights the Ca/Mg ratio’s superior predictive value over absolute levels for periodontal deterioration in at-risk populations.

Complementing these findings, Pushparani et al. [33] performed a case-control study of 600 individuals aged 25 to 56 years, divided into four groups (150 each): healthy controls, type 2 diabetes mellitus without periodontitis, type 2 diabetes mellitus with periodontitis, and non-diabetic periodontitis patients. Serum magnesium was significantly lower in all test groups compared to controls (p < 0.0001), suggesting intertwined roles in diabetes and periodontal susceptibility. Similarly, Raju et al. [34] compared serum magnesium in 55 chronic periodontitis patients and 55 controls aged 18-65, finding lower magnesium in the diseased group (1.57 ± 0.26 mg/dL vs. 1.70 ± 0.27 mg/dL; p < 0.05). Although correlations between magnesium levels and clinical parameters (gingival index, PPD, CAL) were non-significant, the reduction implies magnesium’s influence on disease risk.

In an experimental context, Shetty et al. [35] investigated serum magnesium in 120 participants aged 25-60 across four groups (30 each): chronic periodontitis, type 2 diabetes mellitus without periodontitis, type 2 diabetes mellitus with periodontitis, and healthy controls. After baseline assessments and non-surgical periodontal treatment (scaling and root planing) with follow-up at 21 days, magnesium levels were initially lower in test groups but increased significantly post-treatment (p < 0.05), potentially due to reduced inflammation. Limitations included small sample size, dropouts, short follow-up, and lack of detailed baseline periodontal data, complicating direct links to healing.

Finally, another experimental study by Sundaram et al. [36] in India observed elevated baseline serum magnesium levels in both diabetic and non-diabetic patients with chronic periodontitis three months post-treatment (p > 0.05). This finding offers a contrasting perspective and highlights the need for further exploration into magnesium’s variable responses in comorbid conditions.

Discussion

Overview of Key Findings

The included studies offer compelling evidence of an association between magnesium levels and periodontal health. Despite variations in study designs (observational and interventional), populations, and measurement methods, consistent patterns emerge, emphasizing magnesium’s potential role in mitigating periodontal disease through deficiency or sufficiency mechanisms.

Magnesium and Periodontal Disease Associations

Most studies revealed a significant inverse relationship between magnesium levels and periodontal disease severity. For instance, Meisel et al. [27] and Li et al. [31] showed that higher magnesium intake or serum levels were linked to improved periodontal parameters, such as reduced CAL and PPD, along with lower periodontitis prevalence. Li et al.’s [31] analysis of NHANES data further supported this, demonstrating that increased dietary magnesium correlated with reduced periodontitis risk. These results align with the hypothesis that magnesium exerts anti-inflammatory or protective effects to curb disease progression.

Role of Mg/Ca Ratio

Several studies highlighted the importance of the Mg/Ca ratio, suggesting that magnesium’s interaction with calcium influences periodontal health. Meisel et al. [27,28] and Yoshihara et al. [32] found that lower Mg/Ca ratios were associated with poorer outcomes, including greater tooth loss and attachment loss. This indicates that mineral balance, beyond absolute magnesium levels, is crucial for periodontal maintenance.

Challenges in Measurement Methods

Comparing findings across studies is challenging due to varying magnesium assessment approaches. While many relied on serum or plasma measurements for direct biochemical insights (e.g., Pushparani et al. [33]; Taru et al. [29]; Dannan and Hanno [30]), others used dietary intake for population-level perspectives (e.g., Meisel et al. [27]; Li et al. [31]). These methodological differences may account for variability in reported effects on periodontal health.

Insights from Excluded Studies on Alternative Measures

Although our review prioritized blood-based magnesium levels (serum/plasma) and the Mg/Ca ratio to maintain focus, complementary evidence from excluded studies on saliva and GCF [37-39] offers valuable context on measurement-specific variations. For instance, Baima et al. [39] reviewed salivary ion profiles in periodontitis, reporting mixed results: two studies showed higher magnesium in affected patients, while three indicated lower levels. Romano et al. [37] noted significant differences across healthy, untreated, and treated groups, with untreated patients exhibiting elevated GCF magnesium (mean 8.47 ± 0.77 vs. 6.91 ± 0.78 in controls), reducing to 7.02 ± 0.69 post-treatment. Salivary levels followed a similar pattern (untreated: mean = 19.64 ± 2.01 vs. 13.71 ± 2.32 in controls, decreasing to 14.21 ± 1.86 after therapy) [37]. These suggest periodontal therapy lowers magnesium in GCF and saliva, linking it to inflammation [37,38]. This contrasts with our blood-focused findings of lower magnesium in periodontitis, as tissue breakdown may release magnesium into GCF/saliva despite systemic deficiency [38,39]. Such variations, influenced by disease stage and nutrition, imply that monitoring magnesium changes could aid in evaluating treatment progress.

Magnesium in Systemic Conditions

Studies also examined magnesium’s links to systemic conditions such as diabetes. Pushparani et al. [33] and Shetty et al. [35] reported lower serum magnesium in type 2 diabetes mellitus patients with chronic periodontitis versus controls, indicating synergistic effects. Diabetes, a known periodontal risk factor, underscores the need to consider systemic health in magnesium’s role. Alarcón-Moreno et al. [40] explored magnesium-zinc supplementation with non-surgical therapy in type 2 diabetes mellitus patients, revealing baseline deficiencies and elevated malondialdehyde (MDA), with post-treatment improvements in antioxidant enzymes (superoxide dismutase and catalase), reduced MDA, and better periodontal parameters. However, this study [40] was excluded due to a lack of quantitative post-treatment magnesium data, highlighting the need for standardized reporting.

Causality remains unclear, whether magnesium deficiency worsens periodontitis or inflammation alters magnesium metabolism. Shetty et al. [35] and Sundaram et al. [36] observed improved magnesium levels post-therapy, suggesting inflammation treatment may restore levels and promote healing.

*Mechanisms of Magnesium*’*s Effects*

Magnesium is essential for immune function; deficiency elevates inflammatory markers such as interleukin 6, tumor necrosis factor alpha, and CRP [41,42]. It acts as a calcium-channel blocker, limiting nuclear factor kappa B activation and cytokine production [24,43,44], while low levels impair T-cell function [18,45,46]. These mechanisms are relevant to chronic periodontal inflammation, implying magnesium’s anti-inflammatory properties may protect against deterioration, especially in diabetes [31,47].

Limitations

Despite promising evidence, limitations include small sample sizes, short follow-ups, and lack of randomization, reducing generalizability. For example, Shetty et al. [35] had dropouts and incomplete baseline data, while Pushparani et al. [33] did not assess supplementation’s direct impact on diabetic outcomes.

Future directions

Further research should address these gaps through large-scale, long-term randomized controlled trials to clarify causality and optimal magnesium intake/supplementation. Investigating magnesium’s interplay with calcium and other micronutrients will enhance understanding of its role in periodontal prevention and management.

## Conclusions

This systematic review highlights a consistent association between higher magnesium levels, particularly in balance with calcium, and improved periodontal health outcomes, including reduced disease severity and prevalence. These findings support magnesium’s potential protective, anti-inflammatory role in periodontal disease management. However, methodological heterogeneity underscores the need for large-scale, well-designed clinical trials to confirm causality, establish optimal intake guidelines, and integrate magnesium supplementation or dietary strategies into clinical practice for enhanced patient outcomes.
